# Label-Free Proteomics Assisted by Affinity Enrichment for Elucidating the Chemical Reactivity of the Liver Mitochondrial Proteome toward Adduction by the Lipid Electrophile 4-hydroxy-2-nonenal (HNE)

**DOI:** 10.3389/fchem.2016.00002

**Published:** 2016-03-03

**Authors:** Shin-Cheng Tzeng, Claudia S. Maier

**Affiliations:** Department of Chemistry, Oregon State UniversityCorvallis, OR, USA

**Keywords:** protein carbonylation, 4-hydroxy-2-nonenal, Michael adducts, aldehyde-reactive probe, mitochondria, liver, oxidative stress, Alcoholic liver disease

## Abstract

The analysis of oxidative stress-induced post-translational modifications remains challenging due to the chemical diversity of these modifications, the possibility of the presence of positional isomers and the low stoichiometry of the modified proteins present in a cell or tissue proteome. Alcoholic liver disease (ALD) is a multifactorial disease in which mitochondrial dysfunction and oxidative stress have been identified as being critically involved in the progression of the disease from steatosis to cirrhosis. Ethanol metabolism leads to increased levels of reactive oxygen species (ROS), glutathione depletion and lipid peroxidation. Posttranslational modification of proteins by electrophilic products of lipid peroxidation has been associated with governing redox-associated signaling mechanisms, but also as contributing to protein dysfunction leading to organelle and liver injury. In particular the prototypical α,β-unsaturated aldehyde, 4-hydroxy-2-nonenal (HNE), has been extensively studied as marker of increased oxidative stress in hepatocytes. In this study, we combined a LC-MS label-free quantification method and affinity enrichment to assess the dose-dependent insult by HNE on the proteome of rat liver mitochondria. We used a carbonyl-selective probe, the ARP probe, to label HNE-protein adducts and to perform affinity capture at the protein level. Using LC-MS to obtain protein abundance estimates, a list of protein targets was obtained with increasing concentration of HNE used in the exposure studies. In parallel, we performed affinity capture at the peptide level to acquire site-specific information. Examining the concentration-dependence of the protein modifications, we observed distinct reactivity profiles for HNE-protein adduction. Pathway analysis indicated that proteins associated with metabolic processes, including amino acid, fatty acid, and glyoxylate and dicarboxylate metabolism, bile acid synthesis and TCA cycle, showed enhanced reactivity to HNE adduction. Whereas, proteins associated with oxidative phosphorylation displayed retardation toward HNE adduction. We provide a list of 31 protein targets with a total of 61 modification sites that may guide future targeted LC-MS assays to monitor disease progression and/or intervention in preclinical models of ALD and possibly other liver diseases with an oxidative stress component.

## Introduction

The completion of the human genome project (HGP) in 2003 enabled us to predict the complete repertoire of proteins. During the past decade, proteomic studies have been propelled by the advancement of modern mass spectrometers and related techniques. Hundreds and up to several thousands of proteins can now be detected and identified from biological samples in a single LC-MS analysis routinely (Thakur et al., 2011). However, the analysis of post-translational modifications (PTMs) remains challenging. PTM analysis is complicated due to the multitude of chemically different modifications, the possible presence of positional isomers and that modified proteins only make up a small fraction of the proteins present in a cell or tissue proteome (Jensen, 2006).

The bottom-up proteomics approach is an analytical strategy in which complex protein mixture are first digested to peptides which then are subjected to LC-MS/MS to generate tandem mass spectral data. A database search of the MS/MS spectra assigns them to peptides and reassembly of the information indicates the proteins present in the original mixture. By applying efficient affinity capture tools on top of bottom-up approaches addresses the issue that post-translationally modified proteins are only present at low stoichiometric levels in the whole protein pool, and as a result, lead to significantly increases in sensitivity and specificity (Liebler, 2008).

Nearly 30 years ago, the α,β-unsaturated aldehyde 4-hydroxy-2-nonenal (HNE) was identified as a major product of peroxidation of liver microsomal lipids and found to be toxic to cells (Benedetti et al., 1980). HNE is generated during oxidation of ω-6 polyunsaturated fatty acids which are abundant in phospholipids, the major constituents of biomembranes (Carini et al., 2004). The reactivity of HNE is attributed to three functional groups: conjugated double bond, carbonyl group and hydroxyl group. HNE can react with proteins and other cellular targets which lead to a variety of biological effects including: inhibition of protein and DNA synthesis, inactivation and stimulation of enzymes, resistance to proteolytic degradation and a tendency toward aggregation (Cohn et al., 1996). Proteins and peptides are primary reactants for HNE due to their abundance. HNE reacts with proteins to form predominantly Michael-type adducts involving the nucleophilic side chains of cysteine, histidine and lysine residues (Doorn and Petersen, 2003). HNE-protein adducts have been detected in liver tissues associated with various hepatic diseases and are believed to be a reliable biomarker of lipid peroxidation in liver injury (Browning and Horton, 2004; Begriche et al., 2006; Poli et al., 2008; Serviddio et al., 2011, 2013).

Alcoholic liver disease (ALD) is a progressive inflammatory disease that ranges from steatosis to steatohepatitis, and ultimately leads to fibrosis and cirrhosis (Louvet and Mathurin, 2015). Early pathological mechanisms in ALD have been linked to alcohol-induced oxidative stress-related impairment of mitochondrial function (Bailey and Cunningham, 1998; Cunningham and Bailey, 2001). Chronic ethanol consumption is associated with enhanced mitochondrial ROS production, glutathione depletion and increased lipid peroxidation (Bailey and Cunningham, 1998; Cunningham and Bailey, 2001; Louvet and Mathurin, 2015). Indeed, increased levels of HNE-modified protein adducts have been consistently detected in liver from ethanol-exposed rodents using immunohistochemistry. To this date, only a limited number of studies have become available describing distinct proteins with modification by electrophilic lipoxydation products in ethanol-fed models (Fritz et al., 2011; Smathers et al., 2011, 2013; Andringa et al., 2014).

**Figure 1:**
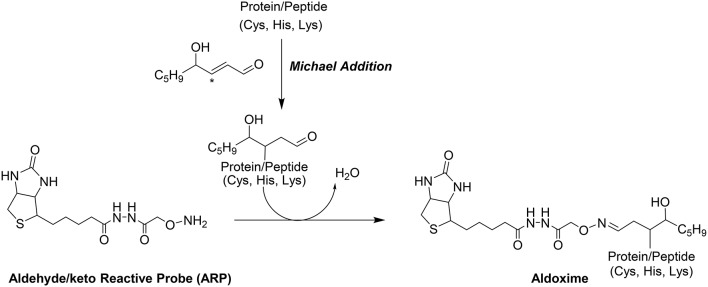
**Modification of proteins by the electrophilic lipid aldehyde 4-Hydroxynonenal, and chemoselective derivatization using the biotinylated hydroxyl amine probe, ARP**.

Shotgun LC-MS methods have been used for profiling the liver proteome in models under induced oxidative stress but these shotgun methods frequently fail to provide the site of HNE modifications (Newton et al., 2009). Our group has introduced a biotin-tagged aldehyde reactive probe (ARP) approach (Chavez et al., 2006, 2010a) that enables the selective derivatization and enrichment of aldehydic peptide adducts in complex biological matrices. The aldehyde functionality of Michael-type conjugates such as HNE adducts, is the target for ARP where it forms a C = N bond to yield a chemically stable biotinylated oxime derivative (Figure 1). The oxime derivative can be detected with both Western blots as well as by mass spectrometry (Chung et al., 2008; Vasil'ev et al., 2014).

**Figure 2:**
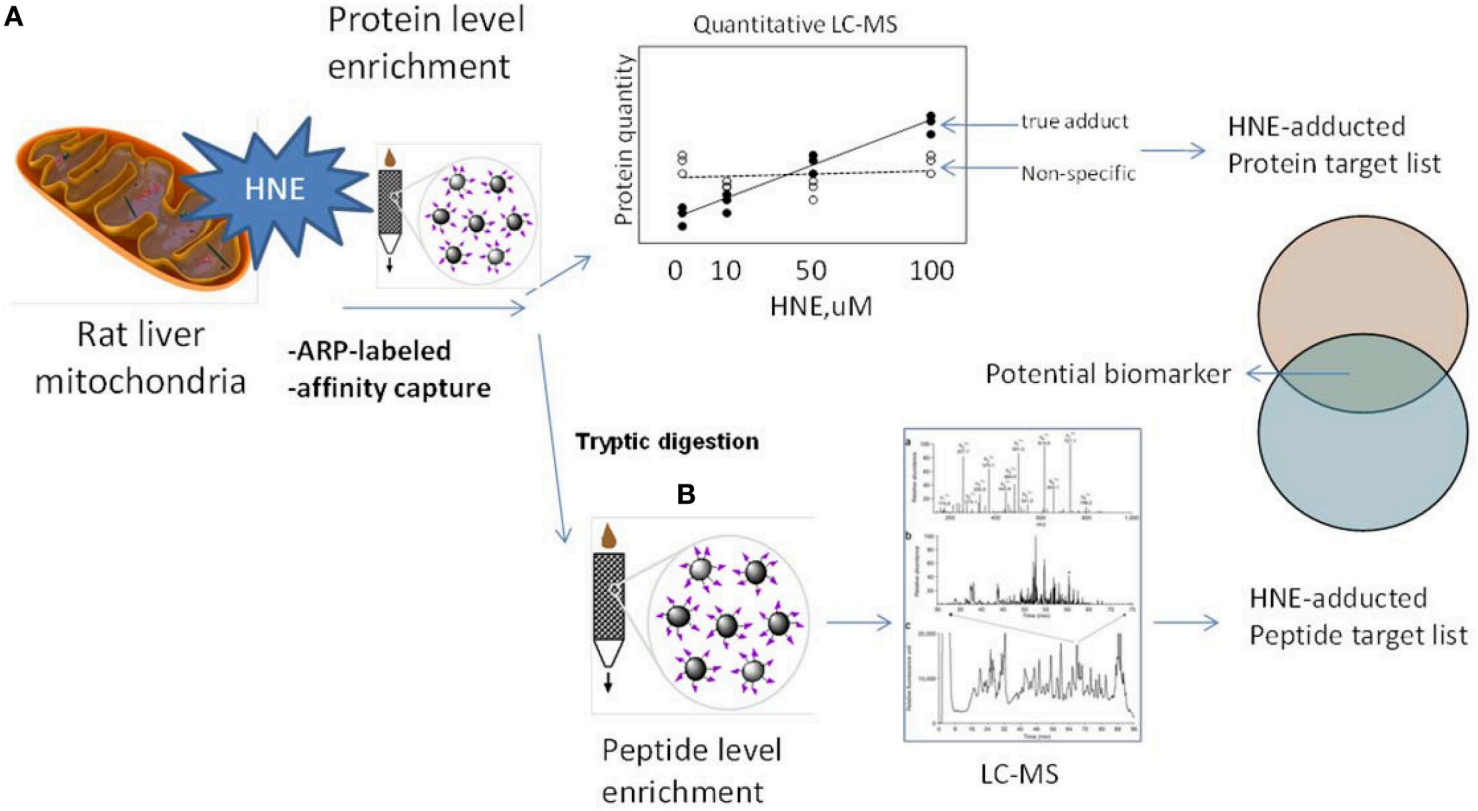
**Experimental design and workflows used in this study**. To explore the protein targets of HNE mitochondrial samples were exposed to HNE, captured, identified, and quantified by implementing two complementary capture strategies: **(A)** at the protein level and **(B)** at the peptide level. We used the ARP probe to label HNE-protein adducts. In strategy **(A)** we performed affinity capture at the protein level. After trypsin digestion, samples were analyzed by label-free quantification approach to identify and quantify protein targets that increase with HNE concentration to obtain a preliminary target list. In strategy **(B)** we applied the affinity capture step at the peptide level, i.e., tryptic digestion of samples were performed prior to the enrichment of HNE-adducted peptides. By this approach, another list of targets with site-specific information was obtained. Those found in both lists were proteins that showed a concentration-dependent response as well as known modification sites. These protein targets of HNE can serve as biomarker candidates useful for future monitoring and/or diagnostic purposes in preclinical models of inflammatory hepatic diseases.

The aim of this study was to complement previous studies by developing a quantitative and sensitive strategy capable of depicting the sub-proteome susceptibility to HNE injury in the hepatic mitochondrion. For this purpose we exposed liver mitochondria to increasing concentrations of HNE (0–2000 μM) to mimic increasing stress status during disease progression. HNE was reported to accumulate up to concentration of 10 μM–5 mM in membranes under conditions of oxidative stress (Uchida, 2003). High concentrations of HNE, such as 500 μM and 2 mM, were included to match conditions of previously reported studies (Uchida and Stadtman, 1993; Carbone et al., 2005; Guo et al., 2011). Figure 2 depicts the workflow involving an affinity capture step at the protein and peptide level in conjunction with label-free LC-MS based proteomic analysis to quantitatively evaluate the concentration-dependent increases in protein adducts. We addressed technical obstacles related to affinity enrichment, assessed kinetics of HNE reactivity and correlated quantitative information from the two enrichment strategies. Overall, the current study portraits the disparate reactivity of the mitochondrial proteome of the liver to the biologically relevant electrophile, HNE. The identified protein targets and sites may serves as candidate biomarkers in future studies designed to determine the functional significance of HNE modification in the different stages of ALD and other hepatic diseases associated with inflammation and oxidative stress.

## Materials and methods

### Materials

Sequencing grade modified trypsin and ProteaseMAX surfactant were purchased from Promega (Fitchburg, WI). Mass spectrometric grade acetonitrile and water were purchased from Honeywell (Morristown, NJ). 4-Hydroxy-nonenal (4-HNE) and Aldehyde-reactive Probe (ARP, N-aminooxymethylcarbonyl-hydrazino D-biotin) were purchased from Cayman (Ann Arbor, MI). Precision Plus protein standard (Dual color) and TCEP (tris(2-carboxyethyl)phosphine) were purchased from BioRad (Hercules, CA). UltraLink-immobilized monomeric avidin, poly-horseradish peroxidase (HRP)-NeutrAvidin conjugate and SuperSignal West Pico Chemiluminescent substrate were obtained from Pierce (Rockford, IL). Nitrocellulose membrane was obtained from Millipore (Bedford, MA). Magnetic streptavidin-coated T1 Dynabeads were obtained from Invitrogen (Carlsbad, CA).

### Rat liver mitochondrial samples

Mitochondrial samples were provided by Dr. Darley Usmar. The preparation procedure was described in Chacko et al. (2011). Briefly, adult male Sprague-Dawley rats were fed with Lieber-Decarli liquid diets for 5 weeks. Liver tissues were harvested at the time of sacrifice. Mitochondria were prepared by differential centrifugation of liver homogenates using ice-cold mitochondria isolation medium containing 0.25 M sucrose, 1 mM EDTA and 5 mM Tris-HCl (pH 7.5). Protease inhibitors were added to the isolation buffer to prevent protein degradation.

### HNE exposure of rat liver mitochondria samples

For preparation of mitochondrial samples for HNE incubation and ARP labeling, 7.5 mg of liver mitochondrial samples were washed twice with 50 mM sodium phosphate buffer (pH 7.4) followed by sonicating at 1 s pulse for 40 cycles using a probe sonicator in an ice box. Triton X-100 was added to give a final 1% solution. The solution was subsequently equally divided into 6 portions for incubation with HNE. Different amounts of HNE stock solution were added to the samples (except for the control group) to obtain the following final HNE concentrations in the solutions: 10, 50, 100, 500, and 2000 μM. Incubation was performed with gentle vortexing at room temperature for 4 h, followed by derivatization with 5 mM of ARP for 1 h. Excess reagents were removed by Centri-Sep gel-filtration spin column from Princeton Separations (Adelphia, NJ).

### SDS-PAGE and western blotting analysis

For gel electrophoresis, 2 μl of sample was add to 10 μl of sample buffer with 50 mM TCEP and incubated in a water bath held at 95°C for 5 min. Samples were then resolved in a 4–12% gel following the protocol as described in Chung et al. (2008). The gel was then transferred to a nitro-cellulose membrane (Millipore, MA) and proteins blotted at 0.15 A for 2 h. The membrane was blocked with 5% non-fat milk in TBS containing 0.1% v/v Tween-20 (TBS-T) overnight.

For detection of ARP-labeled protein bands, the membranes were incubated with 20 ml of 30 ng/mL Poly-HRP-NeutrAvidin for 1 h. After washing the blots six times in TBS-T for 5 min each, the membranes were detected with SuperSignal WestPico chemiluminescent substrate and protein bands were visualized by exposing the membranes to an X-ray film.

### Enrichment of ARP labeled proteins using streptavidin magnetic beads

Enrichment of ARP-labeled samples was achieved by utilizing streptavidin magnetic T1 Dynabeads following vendor's instruction with modifications. Briefly, 100 μl of suspended beads were washed 3 times with 50 mM sodium phosphate buffer (pH 7.4) followed by adding 50 μg of sample and incubated with gentle vortex for 45 min. Beads was separated from unbounded sample on a magnetic stand and washed with phosphate buffer 3 times. To elute ARP-labeled proteins, 50 μl of 0.1% SDS was added to the beads and the suspension briefly incubated in a water bath at 90°C. Beads and solution were immediately separated using a magnetic stand, and the supernatant was carefully taken out. Part of the eluted protein samples were analyzed by SDS-PAGE and Western blotting. The rest of the samples were digested with trypsin utilizing a filter-aided sample preparation method (FASP) (Wisniewski et al., 2009) to remove the detergent for the subsequent LC-MS analyses.

### Peptide level enrichment of ARP-HNE peptide adducts

Affinity enrichment was performed at the peptide level with UltraLink monomeric avidin resin. Samples were tryptically digested and utilized the FASP protocol (Wisniewski et al., 2009) to remove trypsin and other large biomolecules. The digest was then added to a mini spin column packed with monomeric avidin resin. After collecting the flow-through and extensive wash with 1 mL of 2 × PBS followed by an additional wash with 1.5 mL of 50 mM NH_4_HCO_3_ in 20% CH_3_OH to remove non-labeled peptides. The column was then rinsed with 1 mL of MilliQ H_2_O before eluting the captured peptides from the avidin resin by 30% acetonitrile acidified with 0.4% formic acid. The eluate was concentrated using vacuum centrifugation and stored at -20°C before further analysis.

### Reference mitochondrial proteome

A quantitative analysis of unexposed mitochondrial sample was carried out to obtained endogenous protein abundance levels as a reference point for comparison purpose. Briefly, 50 μg of original, unexposed mitochondria were tryptically digested using the FASP protocol as outlined above followed by label-free LC-MS quantification described in the following section.

### LC-MS-based quantitative analysis of enriched proteins

LC-MS analysis of the peptide samples from protein level or peptide level enrichment was performed using a hybrid linear ion trap-Fourier transform ion cyclotron resonance mass spectrometer (LTQ-FT Ultra, Thermo Fisher) coupled to a NanoAcquity UPLC (Waters, MA) equipped with a BEH C18 column (100 μm × 15 cm) (Waters Corp.). A binary solvent system consisting of solvent A, 0.1% aqueous formic acid, and solvent B, acetonitrile containing 0.1% formic acid, was utilized. The sample (2 μl per injection) was automatically loaded onto a Waters Symmetry C18 trapping cartridge (0.3 × 10 mm) and washed for 3 min using 3% acetonitrile containing 0.1% formic acid at a flow rate of 5 μl per min for concentration and desalting. After trapping, peptides were separated on the analytical column at a flow rate of 500 nl/min using the following gradient conditions: (1) 5 min in 97% solvent A for equilibration; (2) linear gradient to 40% solvent B over 25 min and holding at 40% solvent B for isocratic elution for 5 min; (3) increasing the gradient to 90% solvent B and maintaining for 5 min; and finally (4) 97% solvent A for the next 15 min for equilibrating the column. The column was maintained at 37°C during the run. Peptides were electrosprayed using a PicoView nanospray ion source (New Objective, MA). Spray voltage and capillary temperature during the gradient run were maintained at 3.2 kV and 180°C. The mass spectrometer was operated in a data-dependent acquisition mode in which full MS scans from m/z 350 to 2000 were performed in the ICR cell with the resolving power set to 100,000 mass (at m/z 400) using the automatic gain control mode (AGC) of ion trapping. In parallel, tandem mass spectrometry was performed in the linear ion trap mass analyzer on the five most abundant precursors detected in the ICR full MS scan. Peptide fragmentation was induced by collision-induced dissociation (CID) with helium as the target gas. The isolation width was set to 2.0 Da, 35% normalized collision energy with a 30 ms activation time, and an activation Q of 0.25 were utilized. The precursor ion that had been selected for CID was dynamically excluded from further MS/MS analysis for 60 s. Data acquisition was operated with Xcalibur (version 2.2) and Tune Plus (version 2.2) software.

For protein identification and quantification, data were processed with Proteome Discoverer version 1.3 (ThermoFisher, MA) using default parameters. A Mascot search (version 2.3) against Uniprot (http://www.uniprot.org/, 05/12/13,) *Rattus norvegicus* database including common contaminants (28,476 sequences; 13,906,781 residues) was launched from Proteome Discoverer with the following parameters: the digestion enzyme was set to trypsin and two missed cleavage sites were allowed. The precursor ion mass tolerance was set to 5 ppm, while fragment ion tolerance of 0.8 Da was used. Dynamic modifications included carbamidomethyl (+57.0214 Da) for Cys, deamidation for Asp and Gln (+ 0.9840 Da), and oxidation (+15.9994 Da) for Met.

For protein quantification, we utilized the peak integration feature of the Proteome Discoverer 1.3 software. For each identified protein, the average ion intensity of the 3 most intense peptides (Hi3 approach) was used for protein abundance. Scaffold version 3.0 (Proteome Software, Portland, OR) was used for comparative analysis.

### Gene ontology, KEGG pathway analysis, and hierarchical clustering

Mitochondria-related proteins were confirmed by using Gene ontology database (Ashburner et al., 2000) in “cellular compartment” under *Rattus norvegicus*. The proteins identified from protein level enrichment were categorized into 3 groups based on their reactivity profiles. Enriched pathway analyses for each reactivity category were done separately. We used DAVID Bioinformatics Resources (version 6.7) (Huang et al., 2009) to obtained enriched Kyoto Encyclopedia of Genes and Genomes (KEGG) (Kanehisa and Goto, 2000) pathways and a *p*-value for each pathway was obtained. For hierarchical clustering, *p*-values were first log transformed (x = −log_10_ [*p*-value]), and missing *p*-values in the categories were filled in using a *p*-value of 1. Finally, these x values were further transformed to z-scores. The z-scores were then clustered by one-way hierarchical clustering using “Euclidean distance” as distance function and “Average Linkage Clustering” method. Handling of data matrix and heat map visualization were performed with Perseus software (Cox and Mann, 2008).

## Results

### Workflow design

This work presents an analytical strategy (Figure 2) for bridging the gap between discovery-driven research and quantitative applications, such as needed for the validation of potential biomarkers to assist in the elucidation of disease mechanisms. The label-free quantitative LC-MS/MS approach in combination with capture/release strategies enables the identification and quantification of the reactive aldehyde-adducted protein pool in rat liver mitochondria. The study provides a curated set of potential proteins susceptible to modification by reactive lipid peroxidation products that may serve as putative liver disease biomarkers for future quantitative studies using targeted proteomics strategies (e.g., MRM-based proteomics).

### SDS-PAGE and western blot analysis of HNE-exposed mitochondrial proteome

**Figure 3:**
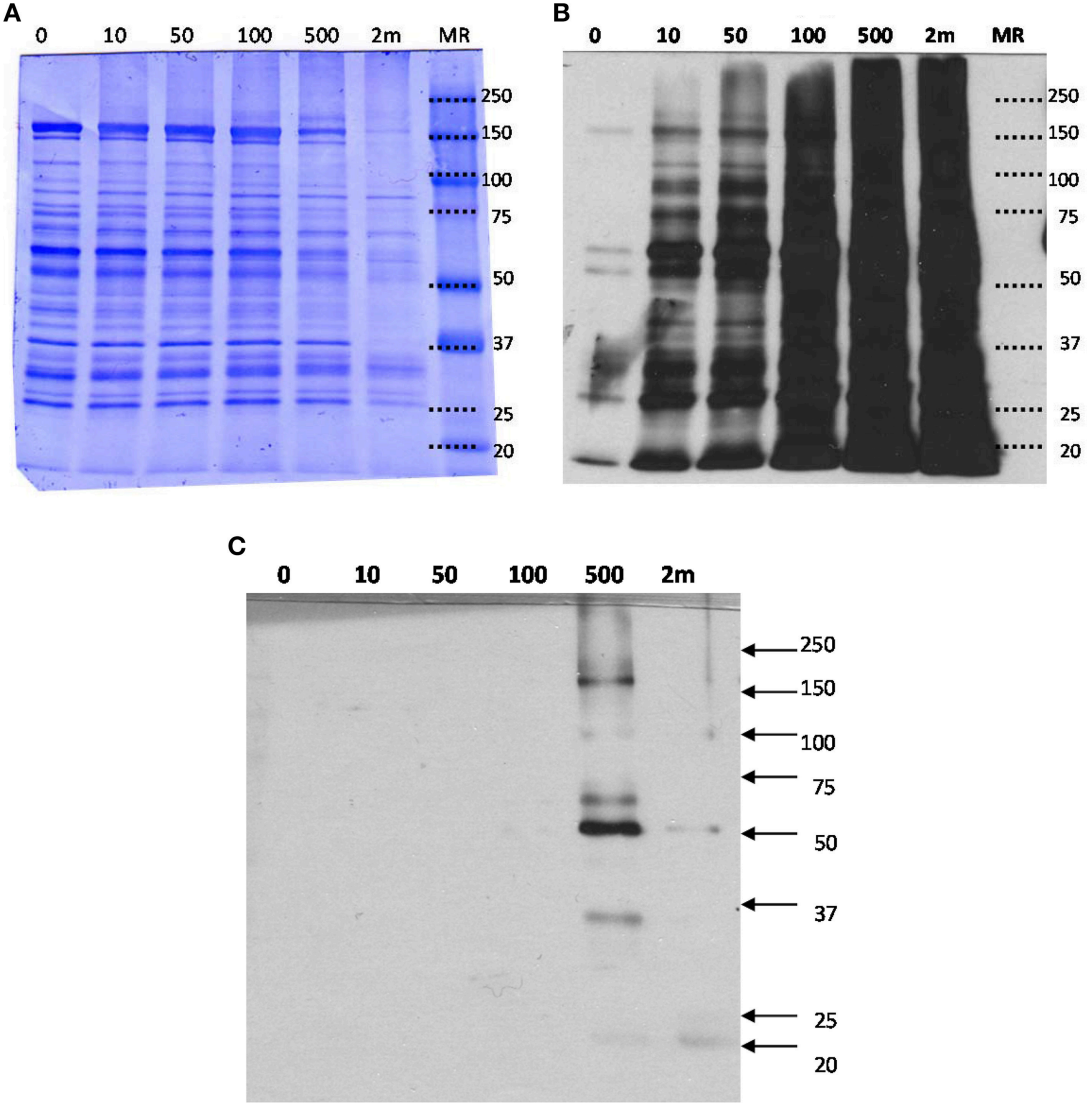
**(A)** SDS-PAGE, **(B)** Western blot analysis of mitochondrial proteins exposed to various concentration of HNE followed by ARP labeling, and **(C)** Western blot with HRP-NeutrAvidin detection of the streptavidin-enriched fractions of the mitochondrial samples. **(A)** HNE-treated mitochondrial protein samples after ARP labeling were analyzed by SDS-PAGE and proteins were stained with Coomassie blue for universal protein detection. **(B)** Western blot analysis with HRP-NeutrAvidin was performed to detect ARP-labeled HNE adducts. **(C)** ARP-labeled mitochondrial proteins were incubated with magnetic streptavidin beads. After extensive washing, captured proteins were released from the beads by elevated temperature in 0.1% SDS. SDS-PAGE and NeutrAvidin blotting of the eluted fractions were performed. There were no visible bands observed for the HNE incubation with concentrations less than 500 μM indicating successful capture of biotinylated proteins. Only for the highest concentration tested (2 mM HNE), bands with weak intensities were visible indicating incomplete affinity capture.

In order to determine the effects of various concentrations of HNE exposure on the mitochondrial proteome, we initially conducted a gel-based study to reveal the presence of HNE adducts in mitochondrial proteins using ARP labeling and Western blotting (Figure 3). Commassie staining showed no visual difference of the protein bands with respect to intensity and pattern among 0–100 μM HNE incubations. However, with higher concentration of HNE (500 μM) treatment, the intensities of the protein bands were slightly reduced, while the pattern of the stained proteins showed no difference. In the extreme case of 2 mM HNE, a significant decrease in staining intensity was observed which potentially indicated occurrence of protein precipitation at this level of exposure. Indeed, unrecoverable protein loss was observed as a consequence of the 2 mM HNE exposure experiment during sample preparation.

To detect ARP-labeled proteins, SDS-PAGE separated proteins were transferred onto a nitrocellulose membrane and biotinylated proteins were detected using avidin affinity staining in combination with chemiluminescence detection (Figure 3B). A dose-dependent increase of HNE adducts was revealed throughout the entire HNE range that was tested (0–2 mM). Several bands (25, 50, 60, and 150 kDa) were observed with visible intensity in the control samples, possibly due to non-specific binding or the presence of endogenous protein carbonyls. Noticeably, intense protein bands were readily observed in the sample treated with 10 μM of HNE before fully saturated signal was observed between 100 and 500 μM. This showed HNE-protein adduction occurred in 10 μM, indicating high reactivity of HNE toward certain proteins (Codreanu et al., 2014).

### Detection by western blotting and LC-MS label-free quantification of enriched ARP-HNE protein adducts

After exposing mitochondria to varied concentrations of HNE, affinity capture of ARP-HNE adducts at the protein level was performed using the aforementioned approach. The pull-downed fractions of all six concentrations (0–2 mM) were analyzed by Western blotting to confirm efficacy of the enrichment. No visible protein bands were observed in Western blots for the 0–100 μM HNE treatments (Figure 3C). Several enriched protein bands were observed in the 20, 35, 50, 60, 100, 170 kDa range in the 500 μM HNE treated sample. As noted previously, the highest concentration of HNE that was used for exposure (2 mM) may have caused substantial protein loss thus showed less adducted protein bands than were observed in the 500 μM HNE exposure experiment.

**Figure 4:**
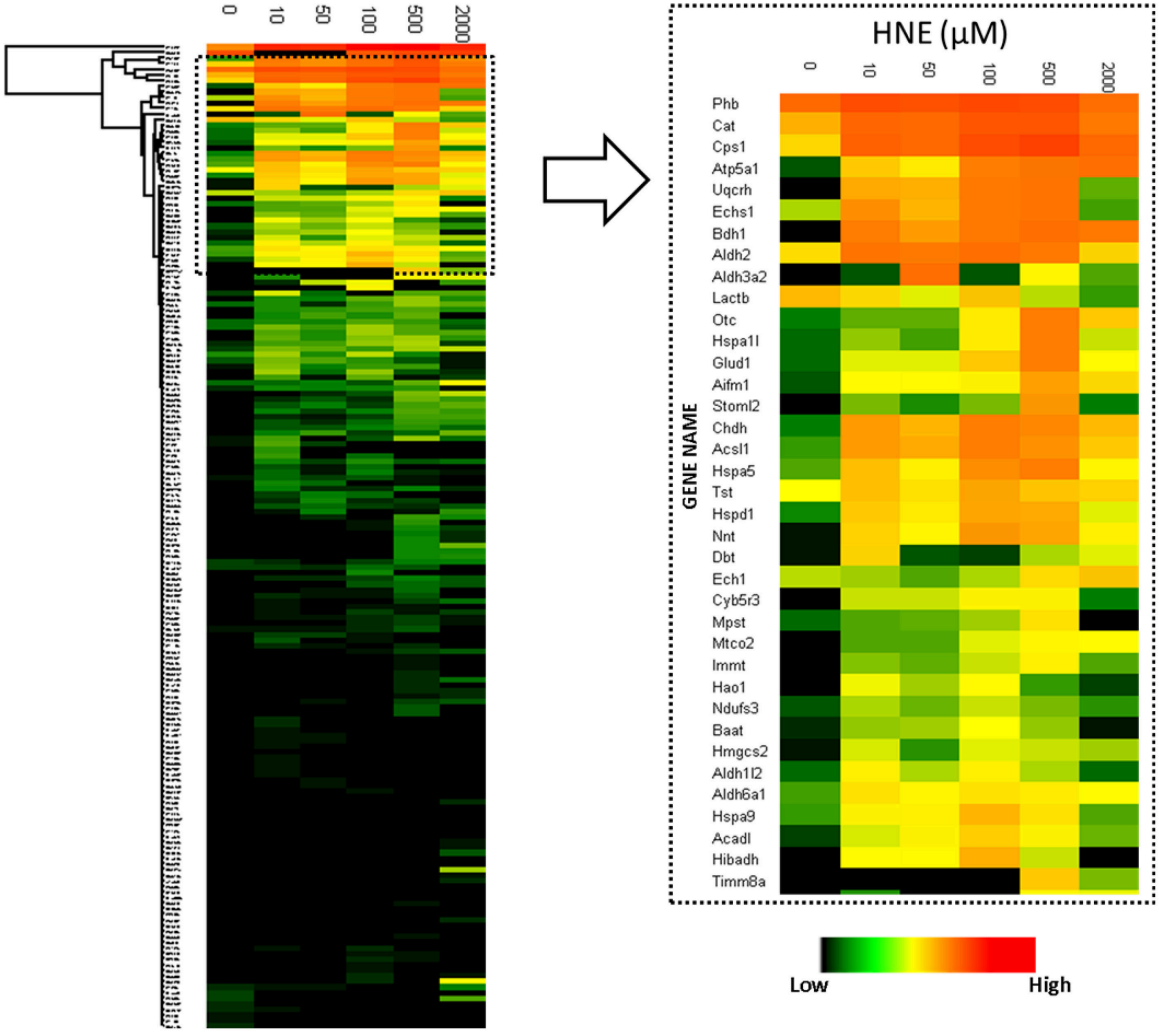
**Heatmap visualization of quantitative values of the 182 proteins enriched at the protein level**. Left: heatmap of the 182 proteins identified and quantified over all six HNE exposure groups; Right: Zoomed-in region of the heatmap focusing on the most abundantly detected and identified putative HNE protein adducts. After affinity capture, samples were trypsin-digested and analyzed using LC-MS. Protein quantification was based on the peak intensity of the 3 most intense peptides. A total of 182 proteins were quantified. From top to bottom the 182 identified proteins are listed and each row represents a protein and its corresponding abundance. From left to right, HNE concentrations that were used for the *in vitro* exposure experiments of the mitochondrial protein samples. The color in each cell represents protein abundance obtained from the “Hi3” peptide intensity approach: red is more abundant and dark green is less abundant. Black indicates missing values.

To reveal concentration-dependent increases in protein adducts with LC-MS-based label-free quantification, enriched samples were trypsin-digested, separated by LC and analyzed on a hybrid LTQ-FT mass spectrometer. A total of 204 proteins were identified and 182 proteins were quantified across all six enriched samples. Figure 4 depicts a heatmap visualization of the levels of protein adducts. Overall, the LC-MS quantification resulted in adduct abundance estimates that were consistent with the Western blot; confirming significant adducts formed at the lowest concentration of HNE exposure (10 μM). Further, the same unexpected decrease of adduct intensity in the sample treated with 2 mM HNE due to protein loss was also revealed.

**Figure 5:**
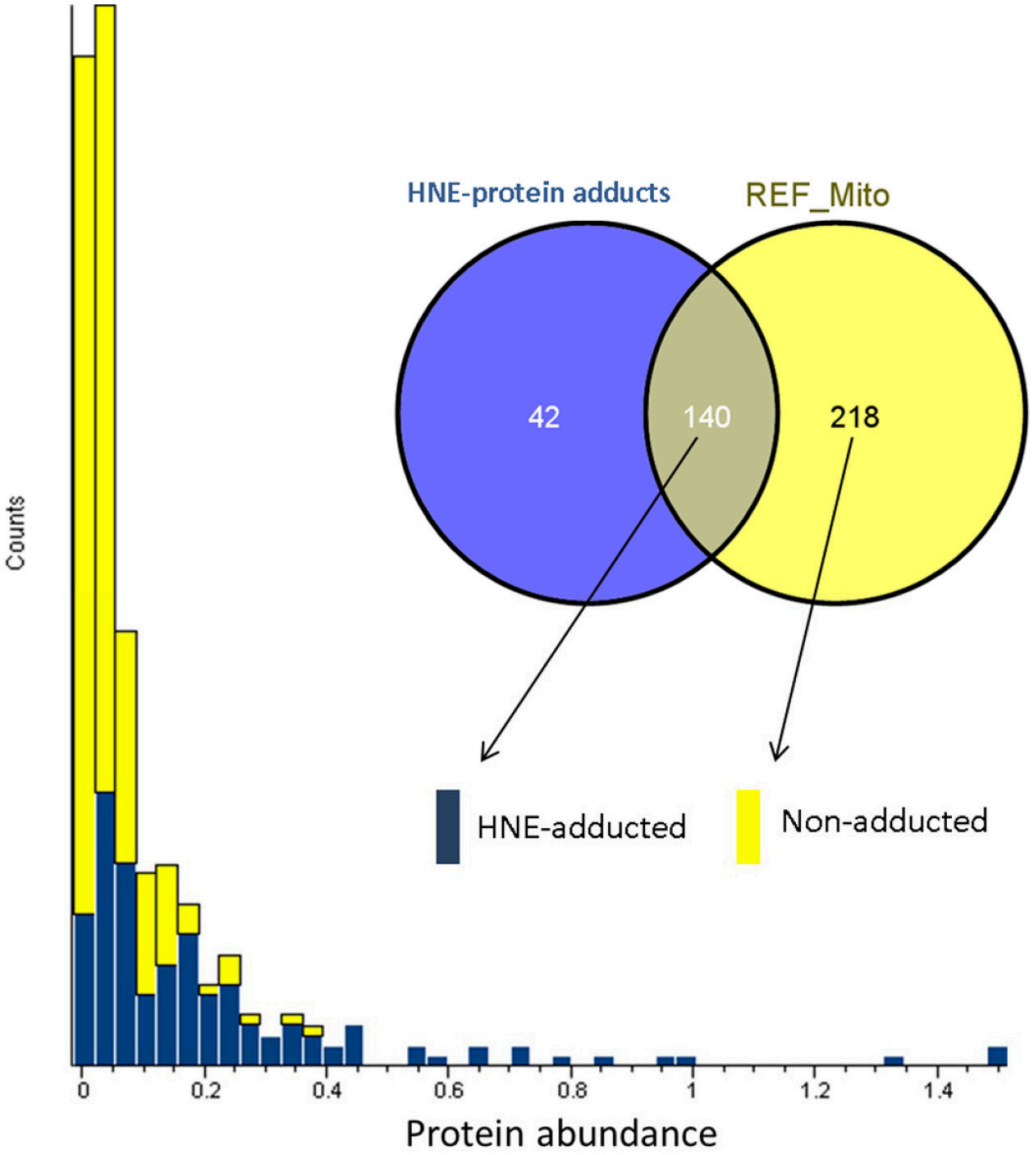
**Abundance distribution of HNE-adducted proteins and non-adducted proteins obtained by profiling the current liver mitochondrial protein preparations**. Protein abundance was obtained from quantifying the “reference” mitochondrial proteome (REF_Mito) that was not exposed to HNE and no enrichment was performed. The yellow bars represent the 218 proteins that were present in the reference proteome. The blue bars show the proteins that were quantified after enrichment and were also identified in the reference proteome (see Materials and Methods section).

To answer whether HNE formed adducts with proteins in a selective fashion or randomly, we compared the abundance of the adducted proteins with the proteins curated in the reference mitochondrial proteome (Figure 5). The comparison showed that all of the highly abundant proteins were also detected as putative HNE adducts. However, a significant number of the low abundant proteins were also found as putative adducts suggesting that HNE reacts with proteins partly according to abundance but also with some degree of selectivity. There were 42 proteins found in our screen that were only identified after affinity enrichment. This implicated the efficacy of the enrichment, and the increased sensitivity of our method for detecting low abundant proteins (as putative targets of HNE) that originally were undetectable.

**Figure 6:**
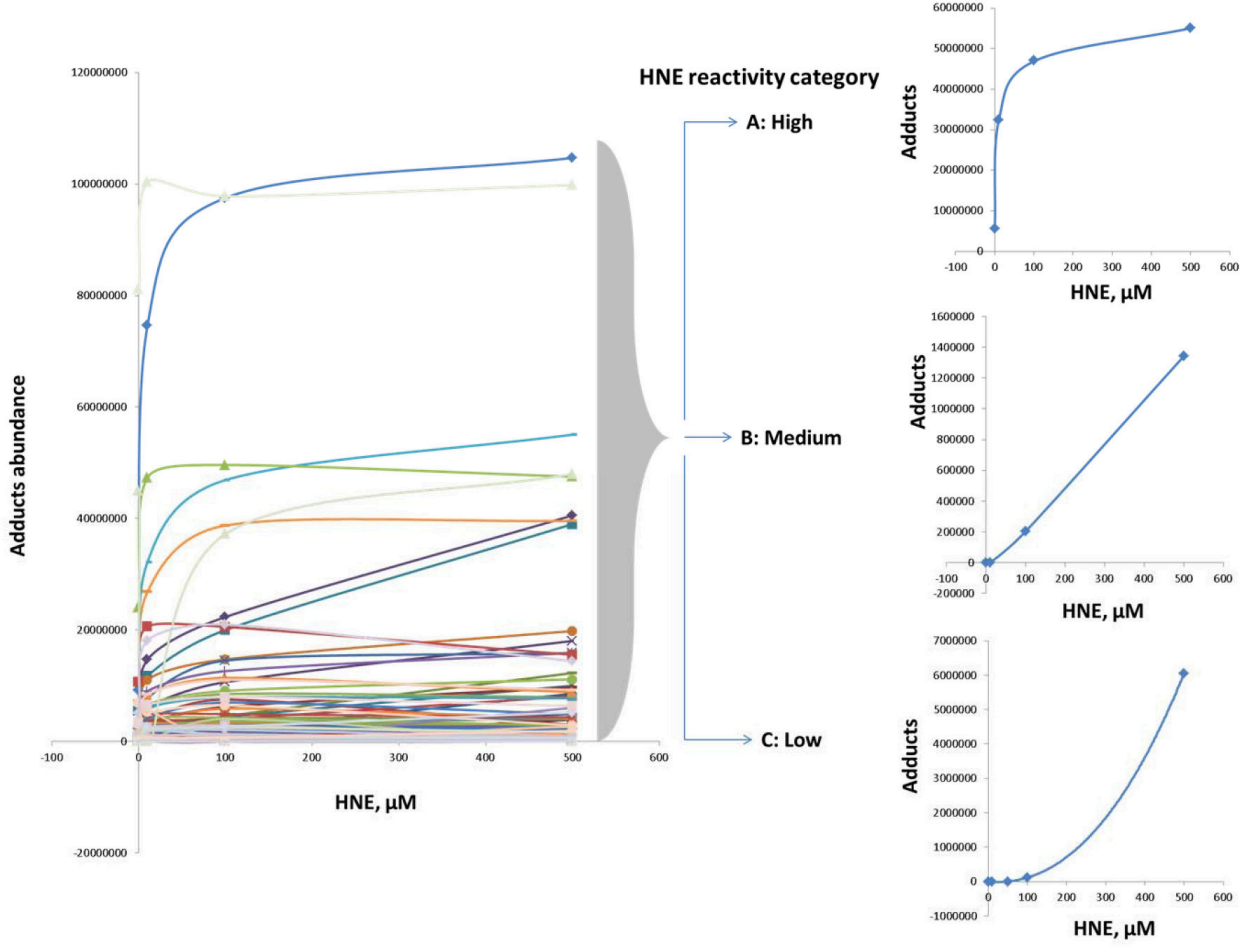
**Categorization according to reactivity based on observed profiles of adduct accumulation in response to increasing HNE concentrations for distinct proteins**. Protein reactivity toward HNE can be grouped into three distinct types: High (category A), medium (category B), and low (category C) reactivity. Category A adducts were formed readily and detected even at low concentration (10 μM). Category B adducts were significantly observed at the medium and high concentrations (50 and 100 μM). Category C adducts were significantly detected only at higher HNE concentrations (>100 μM).

Examination of adduct abundance with increasing concentrations of HNE applied in our exposure experiment suggested that proteins differed significantly in susceptibility to adduction. In order to profile the protein reactivity toward HNE, protein targets of HNE were grouped into three major categories based on the concentration-dependent profile of adduct accumulation (Figure 6). Category A adducts were formed readily even at low concentration (10 μM). Category B adducts were significantly detected at the medium and high concentrations (50 and 100 μM). Category C adducts were significantly found only at higher HNE concentrations (>100 μM). Due to the protein loss caused by precipitation at the highest HNE concentration used in this study (2 mM) we focused our reactivity evaluation only on the results from the 0 to 500 μM exposures.

**Figure 7:**
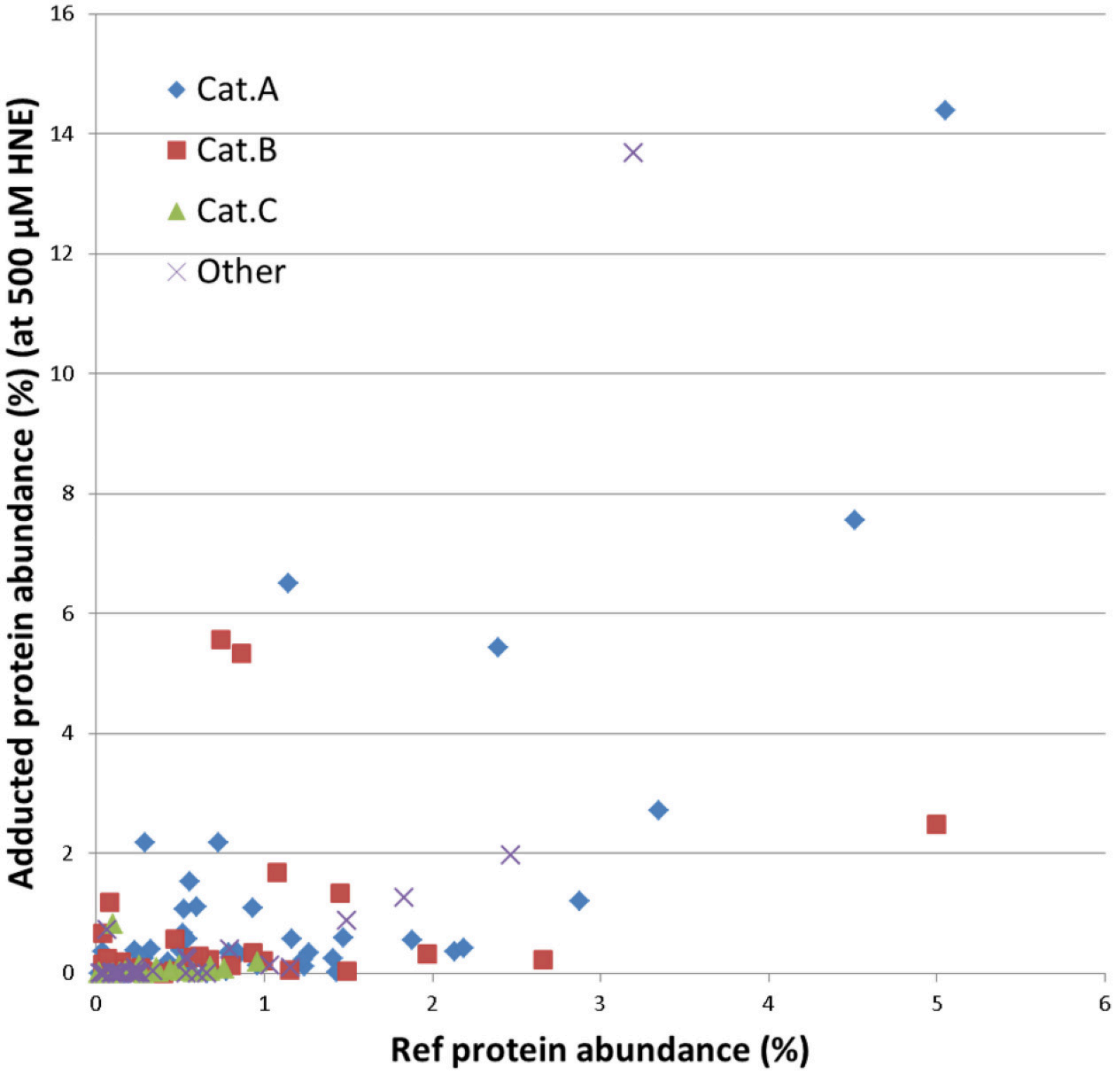
**Correlation of the abundance of the enriched proteins found in the 500 μM HNE exposure experiment with their respective abundance in the reference proteome**. Proteins are marked according to their experimentally observed reactivity toward HNE: Cat A, -highly reactive-; Cat B, -medium reactive-; Cat C, -low reactivity- and other (observed reactivity does not conform to the patterns used for classification; see also Figures 5, 6). This presentation allowed us to cautiously conclude that adduct accumulation seemed to be slightly correlated to the abundance of the respective protein present in the reference proteome. However, Cat A proteins were relatively more abundantly detected in the datasets after enrichment as one would expect from their observed abundance in the reference proteome.

Further, we examined whether the abundance of proteins in our mitochondrial protein preparations may contribute to the observed reactivities with HNE. For this purpose we correlated the abundance of adducts that were assigned from the 500 μM HNE exposure experiment with their abundance observed in the reference proteome. This allowed us to cautiously conclude that adduct accumulation seemed to be slightly correlated to the abundance of the respective protein present in the reference proteome (Figure 7). However, Category A proteins were relatively more abundantly detected in the datasets after enrichment as one would expect from their observed abundance estimates observed in the reference proteome. These observations would be consistent to observations made by us (Chavez et al., 2011) and others (Codreanu et al., 2009, 2014) that proteins show disparate reactivities toward HNE. **Table 2** lists the proteins detected as putative HNE adducts according to their reactivity profiles.

**Figure 8:**
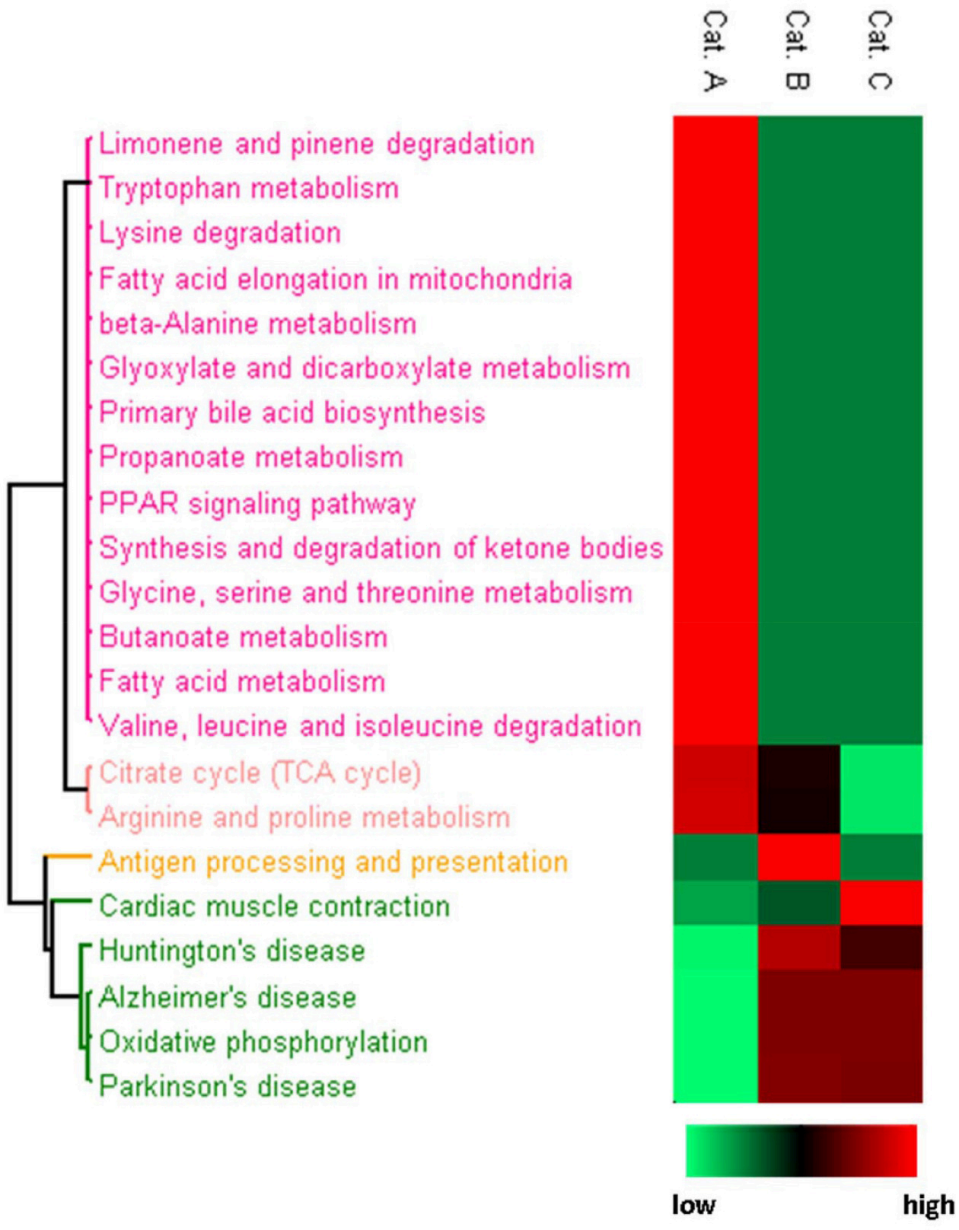
**Heatmap presentation of enriched KEGG pathways for each reactivity category**. Proteins in each category were analyzed separately for enriched pathways. The rows are the enriched pathways for each of the three categories. For the procedure used for generating the heatmap based on *p*-values for the KEGG pathway enrichment in conjunction with Perseus software please refer to the Materials and Methods section. Relative significance is shown in 2-color scheme, red is more significant and green represents less enriched.

Because HNE showed distinct reactivity profiles toward protein adduction, we asked whether this pattern of reactivity could selectively impact functional processes. We used a web-based bioinformatics resource, DAVID (Huang et al., 2007, 2009), to evaluate KEGG (Kanehisa and Goto, 2000) pathway enrichment of protein targets in each category. HNE protein targets in different reactivity categories were enriched for disparately different KEGG pathways (Figure 8). Category A HNE targets were enriched for mainly metabolic processes including amino acid, fatty acid and glyoxylate and dicarboxylate metabolism, bile acid synthesis and TCA cycle. In contrast, Category B and C targets were enriched for oxidative phosphorylation (OXPHOS) and pathophysiological conditions associated with dysfunctional mitochondria (Correia et al., 2012). The findings suggested that OXPHOS proteins were less susceptible to HNE adduction and may imply that the OXPHOS machinery was built to resist oxidative insult in order to preserve its vital function in the cell. Although, the mitochondrial proteins were exogenously exposed to HNE in the present study, our results are in accord with Codreanu and colleagues' *in vivo* cell exposure studies that seem to indicate that OXPHOS proteins seem to be rather resistant to electrophilic aldehyde adduction. They suggested the reduced susceptibility may indicate the evolutionarily adaption of protein systems to protect vital cellular functions (Codreanu et al., 2014).

### Enrichment of adducted peptides for site-specific information

**Figure 9:**
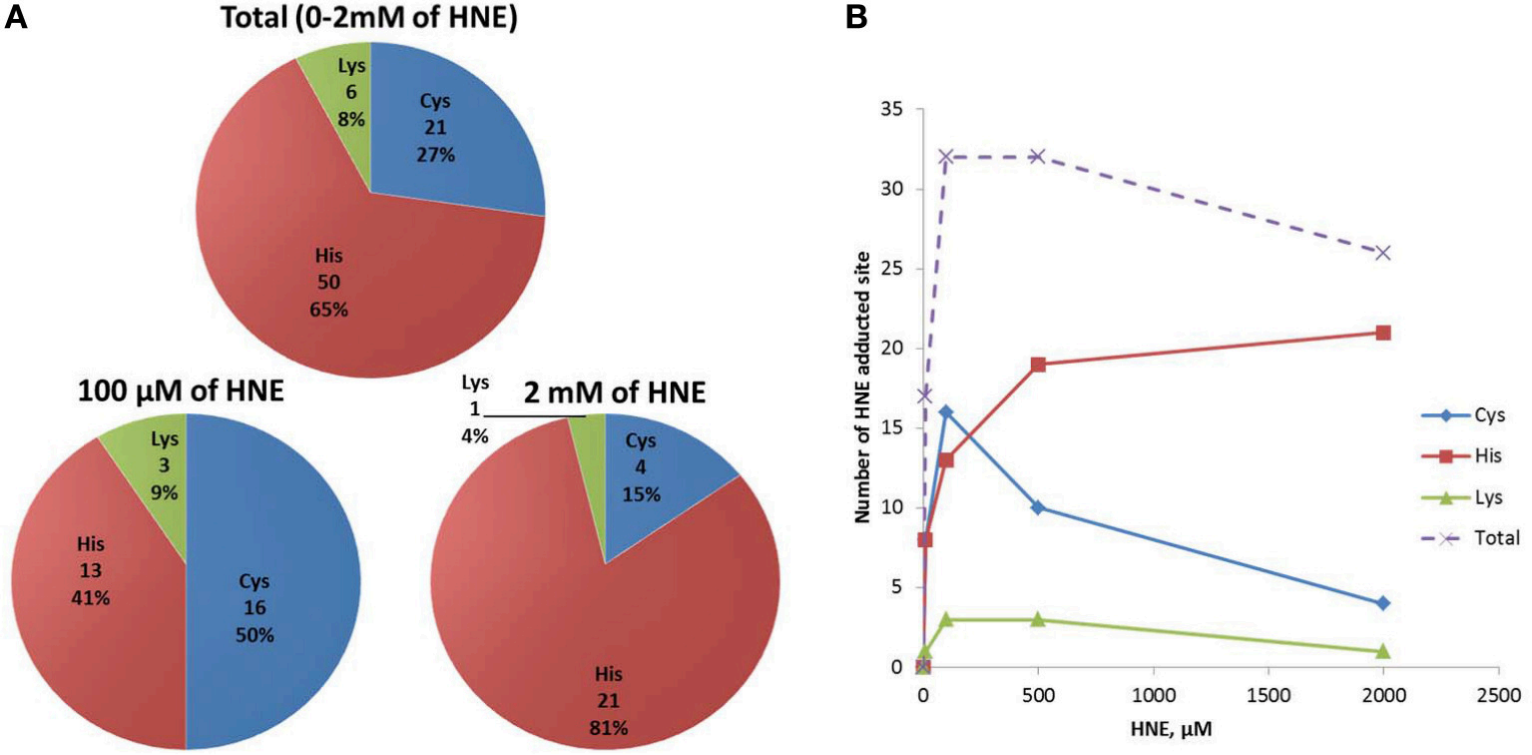
**Numbers of HNE modified Cys, His, and Lys residues identified in HNE exposed mitochondrial samples via peptide level enrichment**. **(A)** Numbers of Cys, His, and Lys HNE adducts identified from the peptide level enrichment analyses combined for all exposure experiments (in total), in the 100 μM and the 2 mM HNE exposure experiments. **(B)** Numbers of Cys, His, Lys adducts as well as total adducts detected in each of the exposure regimes is shown.

To obtain information about HNE-adducted sites at the residue level, a parallel enrichment at the peptide level followed by LC-MS analysis was performed to confirm the presence of the HNE-modification on the proteins and to obtained site specific information. In total, 36 proteins with 77 HNE adducted sites were identified after peptide level enrichment. The reactivity of HNE toward nucleophilic protein side chains has been characterized in the order of Cys>>His>Lys (Doorn and Petersen, 2003). At low concentration (10–100 μM HNE), our result was consistent with that order, as the majority of the adductions were on cysteine residues (50%) followed by histidine (41%) and lysine (9%) (Figure 9A). Our group has observed in a previous study that characterized endogenous 2-alkenal adducts in heart muscle mitochondria from rats using the same ARP enrichment approach, that 85% of the adductions were on cysteine residues, while modifications on histidine and lysine residues were scarce (Chavez et al., 2011). In contrast, at high concentration (500–2000 μM HNE), histidine adducts were dominantly present, and surprisingly, the number of cysteine adducts were lower compared to those from the low concentration exposures (Figure 9B). A similar result was also reported in a study of cytochrome c oxidase (COX) proteins exposed to 2 mM of HNE by Rauniyar and Prokai. They observed most HNE targets were histidine residues, and only one adduction was detection on lysine and one on a cysteine residue (Rauniyar and Prokai, 2009). The deviation from the expected reactivity order may be partly due to protein denaturation and precipitation promoted by the extremely high concentration of reactive aldehyde used. This assumption would be in line with the SDS-PAGE analysis which showed that the protein content was significantly lower in samples treated with 500 and 2000 μM HNE (Figure 3A).

### Comparison of protein level and peptide level enrichment

**Figure 10:**
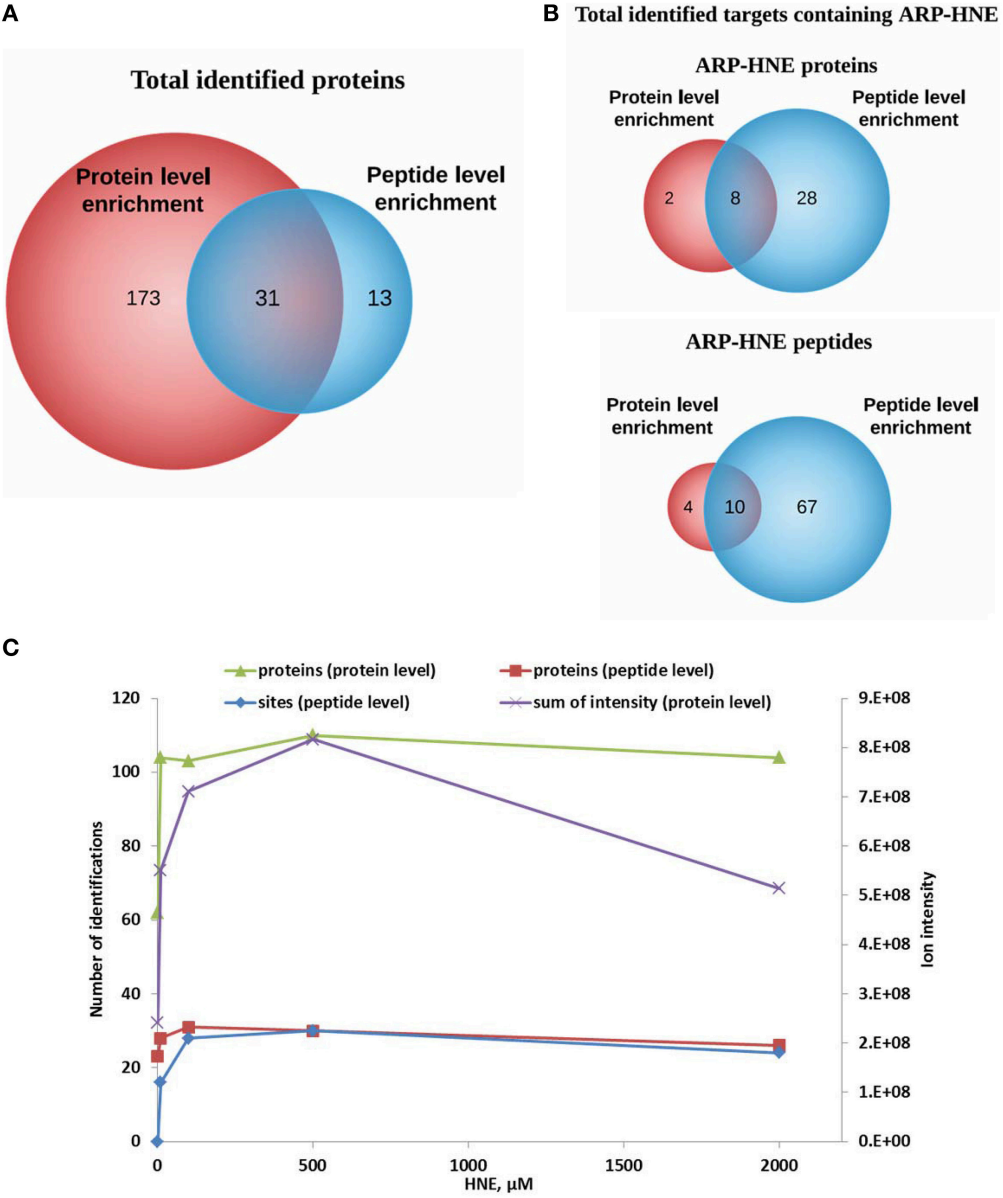
**Comparison of results obtained from protein level and peptide level enrichment in numbers of identified proteins (putative protein targets of HNE identified at the protein level), adducted peptides (proteins identified at the peptide level), and modification sites (at the peptide levels)**. **(A,B)** Venn diagrams of identified proteins and ARP-HNE adducted proteins and peptides from both protein and peptide level enrichment. **(C)**, Plot depicts the numbers of identified proteins, modification sites (peptide level only) and the sum of the peak intensity from LC-MS quantification (protein level only) obtained from samples exposed to varied concentrations of HNE.

A total of 204 and 44 proteins were identified from protein-level and peptide-level enrichment, respectively (Figure 10A). Thirty-one of the 44 proteins identified at the peptide level were also found at the protein level. In terms of adducted site identification, only 4.9% of proteins (10 of 204) were identified with ARP-HNE adducted sites in the protein level enrichment experiment. In contrast, 36 of 44 proteins identified in the peptide-level enrichment experiment were identified with assigned ARP-HNE modification sites. A total of 14 and 77 ARP-HNE peptides were identified from protein level and peptide level enrichment, respectively (Figure 10B). Table 1 presents a compilation of the adducted peptides that were identified.

**Table 1:** **ARP-HNE modified peptide list from the current study**.

**Protein name**	**Gene name**	**Uniprot ID**	**MW**	**Sequence**	**Adducted residue**
ATP synthase subunit beta, mitochondrial	atp5b	ATPB_RAT	56 kDa	(R)LVLEVAQhLGES TVR(T)	His
				(K)AhGGYSVFAGVGER(T)	His
				(R)TREGNDLYhEMIESGVINLK(D)	His
				(R)EGNDLYhEMIESGVINLK(D)	His
				(K)KGSITSVQAIYVPADDLTDPAPATTFAhLDATTVLSR(A)	His
				(R)IMDPNIVGSEhYDVAR(G)	His
				(R)FLSQPFQVAEVFTGHMGk(L)	His
				(K)GFQQILAGDYDhLPEQAFYmVGPIEEAVAK(A)	His
				(K)LAEEhGS(−)	His
D-beta-hydroxybutyrate dehydrogenase, mitochondrial	Bdh1	BDH_RAT	38 kDa	(K)AVLVTGcDSGFGFSLAK(H)	Cys
				(R)TIQLNVcNSEEVEK(A)	Cys
				(K)mETYcNSGSTDTSSVINAVTHALTAATPYTR(Y)	Cys
Voltage-dependent anion-selective channel protein 1	Vdac1	VDAC1_RAT	31 kDa	(K)YQVDPDAcFSAK(V)	Cys
				(K)LTLSALLDGKNVNAGGHk(L)	Lys
				(K)NVNAGGhK(L)	His
ADP/ATP translocase 2	Slc25a5	ADT2_RAT	33 kDa	(K)LLLQVQhASK(Q)	His
				(K)GTDImYTGTLDcWR(K)	Cys
Long-chain-fatty-acid—CoA ligase 1	Acsl1	ACSL1_RAT	78 kDa	(K)ALkPPCDLSmQSVEVTGTTEGVR(R)	Lys
				(R)GIQVSNDGPcLGSR(K)	Cys
				(K)GIAVhPELFSIDNGLLTPTLK(A)	His
Carbamoyl-phosphate synthase [ammonia], mitochondrial	Cps1	CPSM_RAT	165 kDa	(R)SAYALGGLGSGIcPNK(E)	Cys
				(R)SAYALGGLGSGIcPNKETLMDLGTK(A)	Cys
				(R)VSQEhPVVLTK(F)	His
				(R)FLGVAEQLhNEGFK(L)	His
Uricase	Uox	URIC_RAT	35 kDa	(K)DYLhGDNSDIIPTDTIK(N)	His
				(K)NTVhVLAK(F)	His
60 kDa heat shock protein, mitochondrial	Hspd1	CH60_RAT	61 kDa	(K)ISSVQSIVPALEIANAhR(K)	His
				(R)AAVEEGIVLGGGcALLR(C)	Cys
Microsomal glutathione S-transferase	Mgst1	B6DYQ4_RAT	17 kDa	(K)VFANPEDcAGFGK(G)	Cys
				(R)IYhTIAYLTPLPQPNR(G)	His
Catalase	Cat	CATA_RAT	60 kDa	(K)NAIHTYVQAGShIAAK(G)	His
				(R)LGPNYLQIPVNcPYR(A)	Cys
				(R)GPLLVQDVVFTDEMAhFDR(E)	His
				(R)DAMLFPSFIhSQK(R)	His
Choline dehydrogenase (Fragment)	Chdh	Q64644_RAT	49 kDa	(R)KPTQQEAYQVhVGTMR(A)	His
				(K)hELGANMYR(G)	His
				(K)GcPALGDENVPVYKPQTLDTQR(-)	Cys
ATP synthase subunit gamma, mitochondrial	Atp5c1	ATPG_RAT	30 kDa	(R)ThSDQFLVSFK(D)	His
				(K)hLIIGVSSDR(G)	His
3-ketoacyl-CoA thiolase, mitochondrial	Acaa2	THIM_RAT	42 kDa	(K)TNVSGGAIALGhPLGGSGSR(I)	His
				(K)LEDTLWAGLTDQHVk(L)	Lys
Mitochondrial carrier homolog 2	Mtch2	B0BN52_RAT	34 kDa	(K)VLQYYQEcEKPEDLGSANVQK(E)	Cys
ATP synthase subunit alpha, mitochondrial	Atp5a1	ATPA_RAT	60 kDa	(K)hALIIYDDLSK(Q)	His
ATP synthase subunit b, mitochondrial	Atp5f1	AT5F1_RAT	29 kDa	(R)LDYhISVQDMmR(R)	His
				(R)hYLFDVQR(N)	His
				(K)hVIQSISAQQEK(E)	His
Prohibitin-2	Phb2	PHB2_RAT	33 kDa	(R)IGGVQQDTILAEGLhFR(I)	His
Voltage-dependent anion-selective channel protein 3	Vdac3	VDAC3_RAT	31 kDa	(K)ScSGVEFSTSGHAYTDTGK(A)	Cys
				(K)NFNAGGhK(V)	His
Sideroflexin-1	Sfxn1	SFXN1_RAT	36 kDa	(K)hVSPLIGR(F)	His
Glutamate dehydrogenase 1, mitochondrial	Glud1	DHE3_RAT	61 kDa	(K)hGGTIPVVPTAEFQDR(I)	His
ATP synthase subunit d, mitochondrial	Atp5fh	ATP5H_RAT	19 kDa	(K)NcAQFVTGSQAR(V)	Cys
Cytochrome b-c1 complex subunit 2, mitochondrial	Uqcrc2	QCR2_RAT	48 kDa	(R)YENYNYLGTShLLR(L)	His
				(K)NALANPLYcPDYR(M)	Cys
Cytochrome c oxidase subunit 4 isoform 1, mitochondrial	Cox4i1	COX41_RAT	20 kDa	(K)SYVYGPIPhTFDR(D)	His
				(R)DYPLPDVAhVK(L)	His
Bile acid-CoA:amino acid N-acyltransferase	Baat	BAAT_RAT	46 kDa	(K)LTAVPLSALVDEPVhIR(V)	His
Mitochondrial carnitine/acylcarnitine carrier protein	Slc25a20	MCAT_RAT	33 kDa	(K)SVhDLSVPR(V)	His
				(R)LQTQPPSLPGQPPMYSGTIDCFRk(T)	Lys
NADH dehydrogenase [ubiquinone] 1 alpha subcomplex subunit 10, mitochondrial	Ndufa10	NDUAA_RAT	40 kDa	(R)VITVDGNIcSGK(N)	Cys
Nicotinamide nucleotide transhydrogenase	Nnt	Q5BJZ3_RAT	114 kDa	(R)EANSIVITPGYGLcAAK(A)	Cys
Voltage-dependent anion-selective channel protein 2	Vdac2	VDAC2_RAT	32 kDa	(K)SFNAGGhK(L)	His
				(K)ScSGVEFSTSGSSNTDTGK(V)	Cys
Peroxisomal acyl-coenzyme A oxidase 2	Acox2	ACOX2_RAT	77 kDa	(R)SLEDhTPLPGITVGDIGPK(M)	His
Mitochondrial dicarboxylate carrier	Slc25a10	O89035_RAT	31 kDa	(R)GALVTVGQLScYDQAK(Q)	Cys
Pyruvate carboxylase, mitochondrial	Pc	PYC_RAT	130 kDa	(R)LDNASAFQGAVISPHYDSLLVk(V)	Lys
Methylmalonate-semialdehyde dehydrogenase [acylating], mitochondrial	Aldh6a1	MMSA_RAT	58 kDa	(K)GYENGNFVGPTIISNVKPSMTCYk(E)	Lys
Aldehyde dehydrogenase, mitochondrial	Aldh2	ALDH2_RAT	56 kDa	(K)VAFTGSTEVGhLIQVAAGSSNLK(R)	His
Aspartate aminotransferase, mitochondrial	Got2	AATM_RAT	47 kDa	(K)TcGFDFSGALEDISK(I)	Cys
3 beta-hydroxysteroid dehydrogenase type 5	Hsd3b5	3BHS5_RAT	42 kDa	(K)SQSIQGQFYYISDDTPhQSYDDLNYTLSK(E)	His
Very long-chain specific acyl-CoA dehydrogenase, mitochondrial	Acadvl	ACADV_RAT	71 kDa	(R)TGIGSGLSLSGIVhPELSR(S)	His
Ndufa9 protein	Ndufa9	B5DER7_RAT	43 kDa	(K)AVQhSNVVINLIGR(E)	His
Trifunctional enzyme subunit beta, mitochondrial	Hadhb	ECHB_RAT	51 kDa	(R)LNFLSPELPAVAEFSTNETMGhSADR(L)	His
Tricarboxylate transport protein, mitochondrial	Slc25a1	TXTP_RAT	34 kDa	(K)FIhDQTSSNPK(Y)	His
Succinate dehydrogenase complex, subunit C, integral membrane protein	Sdhc	Q641Z9_RAT	18 kDa	(K)NTSSNRPVSPhLTIYR(W)	His
CDGSH iron-sulfur domain-containing protein 1	Cisd1	CISD1_RAT	12 kDa	(K)hNEETGDNVGPLIIK(K)	His
Fatty aldehyde dehydrogenase	Aldh3a2	AL3A2_RAT	54 kDa	(R)FDhILYTGNTAVGK(I)	His

To examine the correlation between the two enrichment methods, results obtained from protein level and peptide level enrichment in numbers of identified proteins (at both levels), adducted peptides (peptide levels only), and total intensity measured by LC-MS analysis (protein level only) were plotted against the HNE concentrations tested (Figure 10C). All four datasets showed positive correlations with increasing HNE concentrations in the range of 0–500 μM. This indicated that the severity of insult with increasing concentration of HNE was revealed by those numbers of putative protein adducts and sites obtained by the two separate enrichment methods and the results were concordant. Notably, the sum of intensities obtained from the LC-MS quantification in the protein level enrichment experiment was lower at the 2 mM compared to the result acquired from the 500 μM HNE exposure experiment. This may be due to protein loss at high concentration of HNE exposure and the finding was consistent with our SDS-PAGE analysis (Figure 3A).

We utilized both protein and peptide level affinity enrichment strategies to generate lists of HNE targets, and combined the two lists to compile a list of proteins that showed a concentration-dependent trend as well as known modification sites. Table 2 compiles the 31 proteins that were found as targets of HNE with both workflows. The proteins are sorted by their occurrence of ARP-HNE sites identified in the peptide level enrichment strategy. The protein with the most adducted sites observed was ATP synthase subunit beta with a total occurrence of 38. In contrast, 11 proteins have 2 or less ARP-HNE peptide occurrences. Linear regression and correlation were applied to evaluate the concentration-dependency of protein abundance on HNE concentration. The upper 27% (54 of 204 proteins) of the proteins found with the protein-level enrichment strategy have regression coefficient values, which represents the slope of the regression line, larger than 1500. Among the 31 proteins that were identified by both enrichment methods, 24 of the 31 proteins fell into the upper region. In accord with the reactivity profile analysis, the five proteins that were found with the most numbers of ARP-HNE peptides identified all belonged to category A, the high reactivity category. As for the correlation coefficient (linearity of the correlation), 21 proteins from the list have linearity larger than 0.617 (whereby a correlation coefficient of 1 represents the perfect linear correlation), which also confirmed the quantitative validity of the list and indicated that the results from the two enrichment methods were comparable. In a separate study conducted by Guo et al., they identified 16 HNE adducted proteins in isolated liver mitochondria upon exogenous HNE exposure using an enrichment approach based on solid-phase hydrazine chemistry in combination with a LC–MS/MS bottom-up approach for identification (Guo et al., 2011). About one-third of their proteins (5) were also revealed in our combined list of the 31 proteins. The five proteins identified by both studies were ATP synthase subunit beta, Carbamoyl-phosphate synthase, 3-ketoacyl-CoA thiolase, Catalase, and Glutamate dehydrogenase 1. The cross-method match further enhanced the validity of the list generated by our approach for its potential use in biomarker discovery and validation studies. The list of putative targets can serve as starting point for further validated studies using orthogonal methods, including high-specificity protein immuno-staining approaches or targeted LC-MS method, to confirm the identity of the protein and further evaluate any concentration-dependent relationships with relevance to biomarker validation studies in various stages of diverse liver diseases.

**Table 2:** **The combined 31 proteins identified in both protein- and peptide-level enrichment workflows**.

**Protein name**	**Gene name**	**Occurrence of ArpHNE peptides**	**Reactivity category**	**Correlation coefficient**	**Regression coefficient**
ATP synthase subunit beta, mitochondrial	Atp5b	38	A	0.627	110812
D-beta-hydroxybutyrate dehydrogenase, mitochondrial	Bdh1	21	A	0.764	27361
Carbamoyl-phosphate synthase [ammonia], mitochondrial	Cps1	12	A	0.729	68122
Long-chain-fatty-acid—CoA ligase 1	Acsl1	12	A	0.352	6051
Voltage-dependent anion-selective channel protein 1	Vdac1	11	A	0.635	311
Microsomal glutathione S-transferase	Mgst1	9	B	0.775	3656
ADP/ATP translocase 2	Slc25a5	8	B	0.981	2993
3-ketoacyl-CoA thiolase, mitochondrial	Acaa2	7	C	0.982	2879
ATP synthase subunit gamma, mitochondrial	Atp5c1	6	B	0.985	521
Catalase	Cat	6	A	0.636	40051
Glutamate dehydrogenase 1, mitochondrial	Glud1	5	B	0.908	14995
Electron transfer flavoprotein-ubiquinone oxidoreductase, mitochondrial	Etfdh	5	B	0.899	2470
ATP synthase subunit alpha, mitochondrial	Atp5a1	4	B	0.910	29111
Prohibitin-2	Phb2	4	B	0.998	4138
Choline dehydrogenase (Fragment)	Chdh	4	other	0.406	7508
ATP synthase subunit d, mitochondrial	Atp5h	4	B	0.917	65516
Trifunctional enzyme subunit beta, mitochondrial	Hadhb	3	A	0.845	1507
Bile acid-CoA:amino acid N-acyltransferase	Baat	3	A	0.277	1366
Sideroflexin-1	Sfxn1	3	B	0.989	3523
Cytochrome c oxidase subunit 4 isoform 1, mitochondrial	Cox4i1	3	C	0.982	1197
60 kDa heat shock protein, mitochondrial	Hspd1	2	A	0.553	7760
CDGSH iron-sulfur domain-containing protein 1	Cisd1	2	C	0.982	285
Aldehyde dehydrogenase, mitochondrial	Aldh2	2	other	0.107	3837
Voltage-dependent anion-selective channel protein 2	Vdac2	2	A	0.217	182
Hydroxymethylglutaryl-CoA synthase, mitochondrial	Hmgcs2	2	A	0.382	2750
Nicotinamide nucleotide transhydrogenase	Nnt	2	A	0.542	9062
Cytochrome b-c1 complex subunit 2, mitochondrial	Uqcrc2	2	A	0.617	1827
Mitochondrial dicarboxylate carrier	Slc25a10	2	B	0.928	2482
ATP synthase subunit b, mitochondrial	Atp5f1	1	C	0.985	1774
Aspartate aminotransferase, mitochondrial	Got2	1	N/A	N/A	0
Succinate dehydrogenase [ubiquinone] flavoprotein subunit, mitochondrial	Sdha	1	other	−0.523	−1758.474

*The list is sorted by total occurrence of ARP-HNE sites. Linear regression and correlation were applied to evaluate the concentration-dependency of protein abundance on HNE concentration; the regression coefficient represents the slope of the regression line and the correlation coefficient represent the linearity of the correlation between adduct accumulation and HNE concentration*.

## Discussion

Our group has developed and applied methods utilizing chemical probes, including ARP, for the analysis of carbonyls in heart mitochondria and THP-1 cells (Chavez et al., 2006, 2010a,b; Han et al., 2007, 2012; Wu et al., 2011). In these previous studies, the enrichment procedure was followed by tryptic digestion, and enabled capture of adducted targets at the peptide level thereby providing information on the type of adduction and the specific site of modification. In this study we explored and evaluated as alternative strategy enrichment at the protein level prior to tryptic digestion. Capturing intact proteins prior to tryptic digestion has several advantages: (1) this strategy will provide greater depth and sequence coverage resulting in more confident protein identifications and quantification compared to workflows in which enrichment is performed at the peptide level; (2) adduction of Lys by HNE (and related 2-enals) causes miscleavages and results in peptide assignments with less confidence; and (3) enrichment at the protein level may provide potential access to protein-protein interaction information (Bildl et al., 2012). Consequently, combining the two enrichment strategies will provide complementary information as more accurate protein quantification is obtained by intact protein enrichment while enrichment at the peptide level results in site-specific annotations. Here we demonstrate the efficacy of the two-prone strategy by determining hepatic mitochondrial proteins that are susceptible to adduction by HNE. We provide information for 31 putative protein targets with a total of 61 unique modification sites including 18 Cys, 40 His and 3 Lys residues. This information may guide future targeted LC-MS assays to monitor disease progression and/or interventions in preclinical models of ALD or other liver diseases.

The avidin (or streptavidin)-biotin interaction is by far the most used affinity capture tool owing to the extreme affinity of native tetrameric avidin for biotin (K_d_ ~ 10^−15^M). Avidin monomers have a much lower biotin-binding affinity than native tetrameric avidin enabling dissociation of biotinylated molecules using mild elution conditions. Chavez et al. (2006) has successfully utilized monomeric avidin for pulling down biotinylated HNE adducts at the peptide level. Adopting the same approach for performing enrichment at the protein level, however, was not as successful, probably due to spatial hindrance caused by the protein resulting in limited access to the monomeric avidin binding site and reduced binding affinity. In this work, we therefore explored the use of magnetic beads coated with streptavidin with high affinity for achieving enrichment of ARP-tagged HNE-adducts at the protein level. However, the strength of the streptavidin-biotin interaction poses challenges for the recovery of biotinylated molecules. To overcome the challenge of reversing the strong binding, we developed and applied a MS-compatible elution approach in the current study. This new approach combines elevated temperature and low concentration of an ionic detergent followed by a detergent clean-up procedure to release, recover and digest captured proteins for downstream Western blot analysis and LC-MS quantification.

Chemoselective probes, such as ARP, are powerful tools but not without limitations. The probe targets protein carbonyls but will also react with other chemical structures that have reactive aldehyde/keto groups. For instance, Chung et al. identified an ARP-reactive protein aspartate aminotransferase which has a covalently linked cofactor with a reactive aldehyde/keto group (Lys259, N6-(pyridoxal phosphate)lysine) (Kirsch et al., 1984). As a result, the protein was detected with avidin staining. In this study, we also identified this protein by both enrichment workflows. However, this protein did not produce enough peptides for quantification which assisted in its assignment as a false positive identification (Table 2, printed in gray).

Using affinity capture in combination with LC-MS label-free quantification, we demonstrated the increase in sensitivity for detecting low abundant proteins that were too low to be detected if unenriched. Advantages of performing enrichment strategies at the protein level are greater coverage, more relevant protein quantification, and specifically, determination of protein isoforms, which can be crucial for biomarker discovery and validation (Zhang et al., 2013). The site-specific adduction information gathered by the peptide level enrichment strategy is not only useful for confirming the putative targets obtained by the protein level enrichment workflow, but also provides valuable site-specific information that can be utilized to generate the transitions of precursor-product ion pairs for developing targeted MRM methods (Gillette and Carr, 2013). For instance, a recent study utilizing a gel-based high-resolution proteomic method for determining targets of HNE in liver mitochondria from chronic ethanol-fed rats describes eight putative protein targets of HNE (Andringa et al., 2014), of which two were also identified in this study, namely choline dehydrogenase and β-hydroxybutyrate dehydrogenase. The current study provides additional information by proposing the modification sites of HNE for the modified proteins, e.g., for choline dehydrogenase proposed modifcation sites are His20, His270, Cys421, and for β-hydroxybutyrate dehydrogenase putative sites susceptible to HNE adduction are Cys63, Cys115, Cys288. However, one should be reminded that the exogenous HNE exposure of mitochondrial proteins may not reflect the actual microenvironment in mitochondria and therefore cannot directly provide information on *in vivo* targets. Nevertheless, *in vitro* exposure studies as described herein do provide a compilation of putative targets that can direct the development of targeted assays for future investigation of the mechanisms associated with chronic ethanol-induced mitochondrial dysfunction and/or possibly other inflammatory liver diseases.

## Conclusion

In this study, a label-free quantitative method in combination with affinity capture for identifying and quantifying HNE adducted proteins in rat liver mitochondria was developed. We employed affinity capture at the protein and peptide level to obtain a complementary list of putative targets of protein HNE adducts for further biological studies. The results from the separate enrichments were quantitatively correlative and complementary. Reactivity categories of HNE targets were revealed by determining concentration-dependent HNE adduction profiles. Proteins that showed high reactivity to HNE adduction were associated with metabolic processes including amino acid, fatty acid and glyoxylate and dicarboxylate metabolism, bile acid synthesis and TCA cycle. Whereas, proteins associated with the ETC displayed retardation toward HNE adduction.

As for reactivity toward different residues, preferred formation of Cys adducts was observed at low HNE concentration, but His adducts were dominant at high concentration.

The study provides sequence information for 31 protein targets with a total of 61 modification sites. The putative protein targets of HNE adduction may serve as leads for further validation using targeted biochemical and/or LC-MS methods to monitor disease progression and/or intervention in preclinical models of ALD and possibly also other liver diseases associated at least in part to altered oxidative stress levels.

## Author contributions

ST carried out the experimental work, mass spectral data generation, data processing and analysis including bioinformatic analysis and data visualization, and prepared the manuscript. CM supervised the study, prepared and edited the manuscript. All authors read and approved the final manuscript.

## Funding

The work was conducted in Oregon State University's Mass Spectrometry Facility which has been supported in part by NIH/NIEHS grant P30ES000210. The development of the chemoselective strategy for tagging protein-HNE adducts was made possible by funds from the NIH/NIA (R01AG025372).

### Conflict of interest statement

The authors declare that the research was conducted in the absence of any commercial or financial relationships that could be construed as a potential conflict of interest.
